# Microfluidics-Based Drying–Wetting Cycles to Investigate Phase Transitions of Small Molecules Solutions

**DOI:** 10.3390/life14040472

**Published:** 2024-04-04

**Authors:** Ajay Verma, Tiphaine Mateo, Juan Quintero Botero, Nishanth Mohankumar, Tommaso P. Fraccia

**Affiliations:** 1 IPGG, CBI UMR 8231—CNRS—ESPCI Paris, PSL Research University, 75005 Paris, France; 2 Department of Chemistry, Columbia University, New York, NY 10027, USA; 3 Department of Pharmacological and Biomolecular Sciences, University of Milan, 20133 Milan, Italy

**Keywords:** microfluidics, chromonic liquid crystals, dry–wet cycles, self-assembly

## Abstract

Drying–wetting cycles play a crucial role in the investigation of the origin of life as processes that both concentrate and induce the supramolecular assembly and polymerization of biomolecular building blocks, such as nucleotides and amino acids. Here, we test different microfluidic devices to study the dehydration–hydration cycles of the aqueous solutions of small molecules, and to observe, by optical microscopy, the insurgence of phase transitions driven by self-assembly, exploiting water pervaporation through polydimethylsiloxane (PDMS). As a testbed, we investigate solutions of the chromonic dye Sunset Yellow (SSY), which self-assembles into face-to-face columnar aggregates and produces nematic and columnar liquid crystal (LC) phases as a function of concentration. We show that the LC temperature–concentration phase diagram of SSY can be obtained with a fair agreement with previous reports, that droplet hydration–dehydration can be reversibly controlled and automated, and that the simultaneous incubation of samples with different final water contents, corresponding to different phases, can be implemented. These methods can be further extended to study the assembly of diverse prebiotically relevant small molecules and to characterize their phase transitions.

## 1. Introduction

The quest to unravel the mysteries surrounding the origin of life on Earth has long captivated the scientific community, leading to an extensive exploration of prebiotic chemistry and the conditions conducive to the formation of life’s building blocks, their assembly, and evolution.

Dry–wet cycles play a pivotal role in the investigation of the origin of life since alternating cycles of hydration and dehydration can simulate the dynamic environmental conditions that early Earth might conceivably have experienced due to day–night and seasonal cycles [1,2,3]. This phenomenon mimics the natural processes occurring in hydrothermal vents, tidal pools, and volcanic environments, all of which are environments where life may have first emerged [4,5].

Dry–wet cycles are instrumental in concentrating and transporting essential building blocks, such as amino acids and nucleotides [6]. Additionally, the importance of dry–wet cycles lies in their ability to promote chemical reactions essential for the formation of complex organic molecules, such as the formation of nucleosides [7], and to eventually drive the self-assembly and the onset of phase transitions in molecular solutions [8,9,10].

Several investigations have shown that during the dry phase, concentrated organic compounds can accumulate and condensation reactions are promoted by the reduced water activity [11]. This is the case, for example, of ribonucleotide polymerization, which has been reported to occur in an acidic pH in the lamellar phases produced by the dehydration of lipids solutions, which is a process that takes the name lipid-assisted polymerization [12,13,14]. Such a process is affected by the depurinization that is caused by a low pH. Recently, the robust polymerization of cyclic 5′-3′ or 2′-3′ phosphate ribonucleotides has been reported in the absence of lipids [15,16,17,18]. Moreover, interaction with mineral surfaces is another crucial process that can further enhance the polymerization of both nucleotides and amino acids [19,20,21].

Drying and heating have also been tested as strategies for the condensation of amino acids and α-hydroxy acids, with findings indicating that in mixtures, amino acids can gradually be incorporated into polyesters, forming depsipeptides, and α-hydroxy acids can favor the formation of amide bonds between amino acids [22,23,24].

Importantly, dry–wet cycles provide crucial features not only during the dry phase. As the wet phase ensues, reacted and unreacted molecular compounds can mix, which is a process that can promote variations by shuffling in the next rounds of polymerization [25]; the wet phase can promote the self-assembly of supramolecular structures, such as lipid and fatty acid vesicles [26,27,28,29,30,31]; and the wet phase can cause phase transitions leading to the formation of complex coacervates [32,33,34,35,36,37] and other membraneless phase-separated droplets [38,39], which can play the role of rudimental protocells.

Molecular concentration and temperature variations during dry–wet cycles can also drive the occurrence of partially ordered still fluid phases that are typical of liquid crystals (LC) in solutions of short DNA and RNA oligomers [40,41,42,43,44,45]. An LC phase transition has been proposed as a relevant physical phenomenon for enhancing nucleic acid polymerization and selection in prebiotic molecular mixtures [8,46,47]. This hypothesis has been supported by the observation of segregation through the LC-phase separation of random sequence oligomers [48,49], and the enhanced non-enzymatic ligation of DNA and RNA oligonucleotides templated by LC ordering in segregative and associative phase separations [50,51,52]. Remarkably, single nucleotides also have the tendency to self-assemble and order in LC phases through a process that involves the hydrogen bonding of nucleobases to form canonical Watson–Crick pairs [53] (in the case of complementary pairs of dNTPs), G-quartets [54] (in the case of guanine rich solutions), or even planar hexagonal structures [55] (in the case of purines and prebiotically relevant base analogs), and undergo successive linear aggregation promoted by π–π stacking of these aromatic rich amphiphilic planar structures. In the field of liquid crystals, such systems take the name of chromonic lyotropic liquid crystals, because they result from the face-to-face aggregation of multi-ring aromatic compounds, typically dyes and drug molecules [56].

Extending the investigation of LC ordering to other nucleotide solutions, including those that are prone to polymerize in dry conditions (such as 5′-3′ and 2′-3′ cyclic nucleotides), can be relevant to unveil phenomena that can potentially provide the optimal geometrical constraints to enhance reactivity in condensation reactions. In this direction of inquiry, it is crucial to explore experimental drying–wetting strategies that allow fine control of the concentration variation and allow the observation of the samples during the process by optical microscopy. Evaporation on glass slides is a well-established method for concentrating lyotropic LC solutions [57] but has several drawbacks, such as the low control of the evaporation velocity, the formation of inhomogeneous regions (e.g., coffee ring effect), and the difficulty of measuring the effective concentration of the sample over time.

For this reason, here we tested microfluidics to create precisely controlled dehydration–hydration cycles of a lyotropic LC-forming aqueous solution of the chromonic dye Sunset Yellow (SSY), which was chosen as the testbed analog of LC-forming nucleotides solutions [56] since its phase behavior has already been quantitatively characterized in previous works [58,59].

## 2. Materials and Methods

### 2.1. Materials

Sunset Yellow FCF (SSY) and chemicals, unless otherwise reported, were purchased from Sigma-Aldrich. Polydimethylsiloxane (PDMS) Sylgard 184 was purchased from Dow and used at a 10:1 prepolymer-to-crosslinker ratio. We used fluorinated oil (HFE7500, 3M) and fluorinated surfactant (FluoSurf-C, Emulseo).

### 2.2. Sunset Yellow Purification and Stock Solution Preparation

SSY was purified from impurities following a previously reported protocol [58,59] consisting of two times repetition of the following: (i) SSY powder dissolution in Milli-Q water, (ii) precipitation by the addition of ethanol, (iii) filtering with a Büchner funnel (VWR) and (iv) drying in vacuum. SSY stock aqueous solutions were prepared by adding the required amount of Milli-Q water to the SSY powder, calculated as v_water_ = m_SSY_/c_0_ − m_SSY_/ρ_SSY_, and considering MW_SSY_ = 452 g/mol and ρ_SSY_ = 1400 g/L, respectively, the molecular weight and the density of SSY.

### 2.3. Microfluidic Chip Fabrication

Single-layer polydimethylsiloxane (PDMS)S chips (~5 mm thickness) were fabricated following standard soft lithography techniques [60]. Microstructured molds were prepared with SU-8 permanent epoxy negative photoresist (micro-resist technology GmbH), following producer processing guidelines and protocol (25 μm height microstructures) on a 100 mm diameter silicon wafer (Darwin microfluidics). A 10:1 ratio of Sylgard 184 PDMS (~55 g) was poured on the SU-8 silicon wafer mold, degassed in a vacuum chamber, and baked at 65 °C for 1 h in an oven. The cured PDMS was then peeled off of the wafer, cut out, and access holes were punched using a stub. The PDMS slab was finally bonded to a standard microscope glass slide (1 mm thick) with an oxygen plasma treatment (10 W for 30 s at 150 mT O_2_), which was followed by overnight incubation at 90 °C.

For double-layer PDMS chips, a thick layer (~5 mm) and a thin PDMS membrane (100 μm) were fabricated separately and then bounded together by oxygen plasma surface activation, which was followed by overnight incubation at 90 °C. PDMS membranes were produced by spin coating a 10:1 ratio of Sylgard 184 PDMS (750 rpm for 40 s, ramp rate of 200 rpm/s) on a SU-8 Silicon wafer mold previously treated with trichloro (1H,1H,2H,2H-perfluorooctyl) silane in the gas phase.

### 2.4. Microfluidic Chip Operation and Microscope Image Acquisition

Three microfluidic chips were used. Chip 1, a single-layer PDMS chip, was used to study the phase diagram of SSY droplets upon evaporation (Supplementary Materials). Chip 2, a double-layer PDMS chip, was adapted from ref. [61] (upper layer) with the addition of a bottom layer with a reservoir channel and was used to perform dehydration and rehydration cycles (Supplementary Materials). Chip 3, a double-layer PDMS chip, was adapted from ref. [62] and was used to incubate SSY solutions at different final concentrations (Supplementary Materials).

The microfluidic chips were plugged with Tygon tubing (1/16” OD × 1/32” ID, Darwin Microfluidics) to a pressure controller for microfluidics (MFCS-EZ, Fluigent) managed by computer software (Maesflo v3.1, Fluigent). The following pressures were applied to the chip inlets for device operation: chip 1: oil pressure = 100 mbar, SSY pressure = 50 mbar; chip 2: oil pressure = 190 mbar, SSY pressure = 200 mbar, reservoir pressure = 300 mbar for exchanging the solutions (water and salt), then 10 mbar during the experiment; chip 3: oil pressure = 200 mbar, SSY pressure = 200 mbar, reservoir pressure = 50 mbar. In chips 1 and 2, the first droplets, initially produced during pressure equilibration, were discarded by letting them exit the chip. After droplets production (chips 1 and 3), the oil pressure was gradually reduced to facilitate trapping.

SSY droplets were observed with an inverted polarized optical microscope (Ti-U, Nikon). Bright-field and crossed polarizer images were acquired at constant time intervals, using 10× or 20× ELWD objectives, a Nikon DS-Fi3 color camera, and NIS Element AR (version 5.30.03) acquisition software. Temperature, T, was controlled by a Peltier hot/cold stage for microscopy (TSA12Gi, Instec Inc., Boulder, CO, USA).

### 2.5. Image Analysis

Images were initially analyzed with ImageJ (version 2.14.0/1.54f, http://imagej.net (accessed on 7 July 2023)) software and then with a custom Python (version 3.11.4) code (Supplementary Materials) properly realized to (1) identify droplets from bright-field images, generate enclosing masks by circular fitting, measure the diameter of the droplet for each time step; (2) measure the average intensity of polarized images, I_p_, within the corresponding circular masks for each time step; (3) calculate the droplets volume and SSY concentration for each time step (see Appendix A); (4) identify the frames corresponding to the phase transitions; and (5) calculate the concentrations corresponding to the phase transitions.

## 3. Results

Inspired by the fact that the permeation of water through PDMS has been exploited to produce microfluidic devices capable of controlling and measuring the concentration of aqueous solutions inside microdroplets [63], here we investigated if the phase behavior of SSY solutions could be measured and controlled with microfluidics. Microfluidic devices have been used to investigate the phase diagram of salt–polymer mixtures or to finely control protein crystallization temperature and concentration conditions [62]. Additionally, it has been shown that concentration-dependent phase transitions leading to the LC ordering of surfactants or fd virus solutions can be achieved by controlled water evaporation in microchannels [64] or in larger volumes [65], respectively. It has been also shown that in PDMS chips, the microchannels’ surface wettability, geometry, topography, anchoring, and other features can spatiotemporally tune phase transitions of solutions of the lyotropic chromonic dye disodium cromoglycate (DSCG) [66,67].

On the other hand, lyotropic liquid crystal droplets, including chromonic SSY and cellulose nanocrystals, have been produced in water-in-oil emulsion [68,69], and osmotic droplet shrinkage has been exploited to observe phase transitions or topological reconfigurations [70].

Here, we tested three microfluidic devices to measure the concentration–temperature phase diagram of SSY solution (chip 1), to realize drying–wetting cycles (chip 2), and to incubate SSY solutions at different final hydration states, corresponding to different phases, in parallel (chip 3).

### 3.1. Measurement of the SSY Phase Diagram

**Figure 1:**
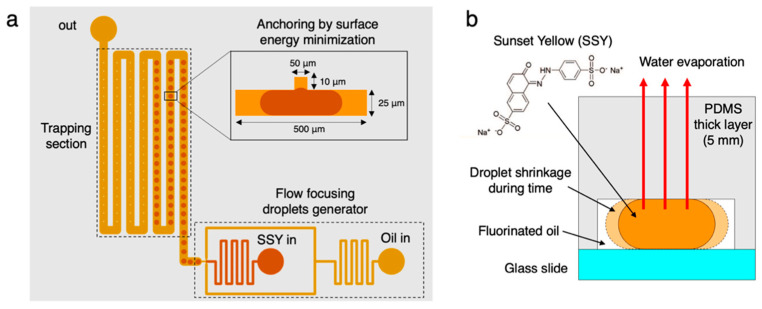
Sketch of the microfluidic chip 1: (**a**) including a flow-focusing droplets generator and the trapping section, which is obtained by droplets anchoring to a hole in the channel roof due to surface energy minimization. A side view of the traps is reported in the inset (not in scale). (**b**) Scheme of the water evaporation process through the PDMS layer exploited to increase the c_SSY_ in the trapped droplets.

We first tested if the phase diagram of the chromonic dye SSY could be measured in microdroplets undergoing slow evaporation in a PDMS microfluidic device, which is sketched in Figure 1a. Aqueous isotropic SSY droplets, at an initial concentration c_SSY,0_ = 0.33 M, in fluorinated oil with 2% surfactant, were produced by a flow-focusing junction integrated into the microfluidic PDMS chip 1. Droplets were trapped by anchoring to circular holes (d_T_ = 50 μm, h_T_ = 10 μm) in the channel roof due to surface energy minimization [71]. The height and the width of the rectangular section channel were h = 25 μm and l = 500 μm, respectively. Water evaporation through the PDMS layer over time caused the shrinkage of SSY droplets and the consequential increase in c_SSY_ (Figure 1b).

**Figure 2:**
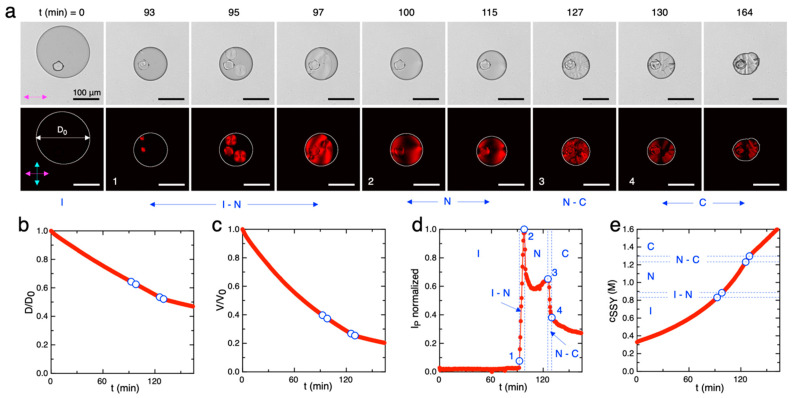
Measurement of the concentration phase diagram of SSY solutions during drying at T = 25 °C. (**a**) Bright-field (top) and polarized (bottom) microscopy images of an SSY droplet trapped in the microfluidic chip and undergoing phase transitions during evaporation. Numbered frames are the ones corresponding to the phase transitions I to I–N (1), I–N to N (2), N to N–C (3), and N–C to C (4). Pink and light blue arrows indicate the directions of the polarizer and analyzer, respectively. Scale bars are 100 μm. Graphs reporting the time evolution of the measured normalized droplet (**b**) diameter D/D_0_, (**c**) volume V/V_0_, (**d**) polarized intensity I_P,_ and (**e**) SSY yellow concentration c_SSY_. Blue open circles and dashed lines indicate the phase transition points between the isotropic (I), isotropic–nematic coexistence (I–N), nematic (N), nematic–columnar coexistence (N–C), and columnar (C) phases.

In a typical experiment performed at room temperature, the images acquired during time (Δt = 60 s) in bright field and polarized microscopy (Figure 2a) showed that as the volume of the initially isotropic (I) phase droplets gradually decreased, birefringent domains with the typical textures of the nematic (N) LC phase started to nucleate, grow and coalesce until the droplets became completely occupied by the N phase. Subsequently, the N phase was stable for a certain amount of time until columnar (C) phase domains started to nucleate and pervaded the whole volume. Finally, the droplets lost their smooth circular shape, which is a sign that the columnar phase was losing its fluidity and turning crystalline.

Bright-field image analysis by ellipse fitting allowed the measurement of the diameter of the droplet, D (Figure 2b), and the calculation of the droplet volume, V, as a function of time (Figure 2c and Appendix A). The observation and the measurement of the polarized image intensity, I_p_, over the droplet area allowed the identification of the phase transition time points (numbered open circles), as reported in Figure 2d. Specifically, the beginning of the I–N coexistence (point 1) was identified as the first frame in which birefringent N phase domains were present, corresponding to I_p_ greater than the background; the end of the I–N coexistence (point 2) was identified as the frame in which the birefringent N textures occupy the whole droplet, corresponding to the maximum of I_p_; the beginning of the N–C coexistence (point 3) was identified as the frame in which columnar domains start to nucleate inside the N textures, generally corresponding to a local maximum of I_p_; the end of the N–C coexistence (point 4) was identified as frame in which columnar textures occupy the whole droplet.

Finally, the SSY concentration was calculated by assuming mass conservation as c_SSY_ (t) = c_SSY,0_ × V(t)/V(t_0_), and the concentrations corresponding to the phase transitions were identified (Figure 2e).

**Figure 3:**
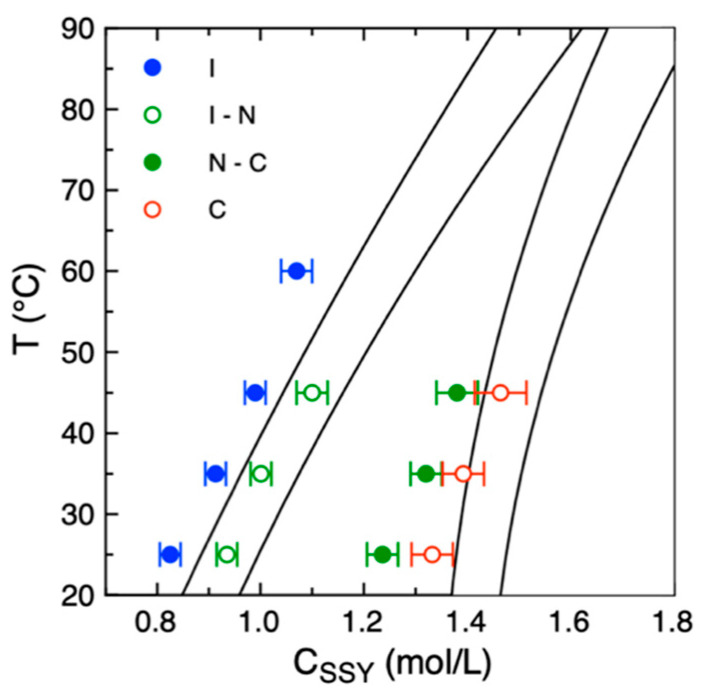
Concentration–temperature phase diagram of SSY solutions obtained by drying droplets within the microfluidic chip (symbols) compared with the one reported in ref. [59] (solid lines). Data points and error bars are reported as the average and standard deviation of N = 5 measurements performed on different droplets.

The SSY concentration–temperature phase diagram was then sampled by repeating the procedure at different temperatures, T = 25 °C, 35 °C, 45 °C and 60 °C. At T = 60 °C, the high velocity of water evaporation allowed us to measure only the I to I–N transition with enough accuracy. The obtained phase diagram is in agreement with the ones reported in the literature [58,59] even if the transition concentration values are slightly underestimated (Figure 3). The difference between the expected and measured values is of the order of 5–10%. Such discrepancy can be attributed to a systematic error in the measurement of the initial volume of the droplets, V_0_, which can already be affected by evaporation during the operations of droplets production and trapping. Indeed, the measured volume decrease after the first 5 min is of the order of 5–6% at 25 °C (Figure 2c). This fact indicates that the time interval between the production of the droplets and the beginning of image acquisition is a crucial parameter that should be minimized to perform a more accurate measurement of the concentration. For this reason, we discarded experiments in which the image acquisition of the trapped droplets started after 5 min from the initial fluxing of the SSY solution in the microfluidic device.

### 3.2. Implementation of Dry–Wet Cycles in the Microfluidic Device

**Figure 4:**
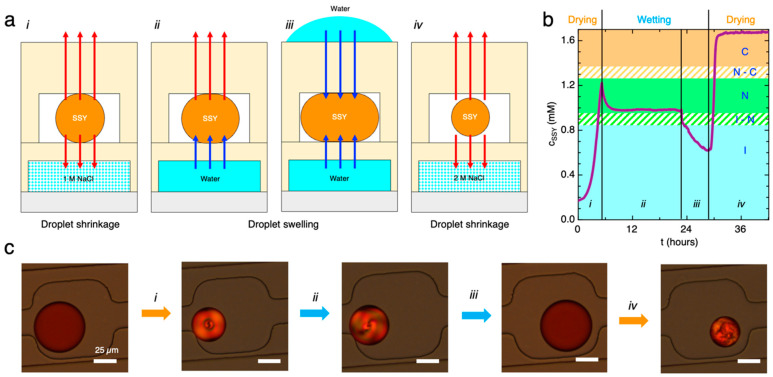
Dry–wet cycles in the microfluidic device. (**a**) Sketches of the dehydration (i and iv) and rehydration (ii and iii) steps obtained by exchanging the flux in the reservoir channel (NaCl or water) and modifying the evaporation from the top PDMS layer. Red and blue arrows indicate the direction of the water transport. (**b**) Variation of c_SSY_ inside a droplet during time calculated from the measured droplet volume change. Vertical solid lines identify the different drying and wetting phases of the cycle. The colored background identifies the phase diagram of the SSY solution inside the droplet. (**c**) Partially de-crossed polarizers microscopy images of the SSY droplets during the dry–wet cycle showing droplet shrinkage and swelling and the transitions between the different phases.

We then tested the possibility of reversibly inducing phase transitions in the droplets trapped in the microfluidic chip 2 by dehydration–hydration cycles. For this purpose, we adopted a double-layer microfluidic chip with a bottom layer containing a reservoir channel (height of the reservoir channel h_r_ = 50 μm), placed underneath the droplet traps and separated by a thin PDMS membrane (h_m_ = 50 μm), in which NaCl or water solutions can be fluxed to osmotically drive droplets shrinkage or swelling (Figure 4a). In the upper layer, the traps consisted of smoothed angles squared chambers with higher height than the main channel, which was designed to trap droplets by surface tension relaxation [61]. The height of the main channel was h_c_ = 20 μm and that of the rectangular traps was h_t_ = 35 μm.

To remove water from the SSY droplets, the reservoir channel was first loaded with 1 M NaCl solution (i). Water transport through the PDMS membrane and evaporation from the top PDMS layer induced the shrinkage of the SSY droplets and their transition to N phase (Figure 4b) as a consequence of the c_SSY_ increase, which was calculated from the volume variation (Figure 4c) as explained in the previous section. Subsequently, water was fluxed in the reservoir channel (ii) to invert the osmotic potential between the reservoir and the SSY droplets. We observed droplets swelling but not the transition to the initial isotropic state. Indeed, the droplets dimension and c_SSY_ reached a plateau value caused by a balance between hydration from the bottom channel and evaporation from the upper PDMS layer. The addition of water on top of the microfluidic chip (iii) contrasted evaporation and allowed further wetting of the droplets and their consequent transition to a lower concentration isotropic phase. To induce drying again, we substituted water with 2 M NaCl solution in the reservoir channel, and we removed the water from the top of the chip (iv). The SSY droplets concentrated faster than the first dehydration step due to the higher osmolarity of the salt solution and transitioned to the N and C liquid crystal phases. This indicates that different salt concentrations in the reservoir channel can be exploited to tune the velocity of the drying process and the final equilibrium phase. Since the timescale of the drying process was of the order of hours, no appreciable effects on the nucleation and growth of the N phase or in the subsequent transition to the C phase or in the LC textures were observed.

### 3.3. Incubation of SSY Solutions in Different Phases by Controlled Drying

**Figure 5:**
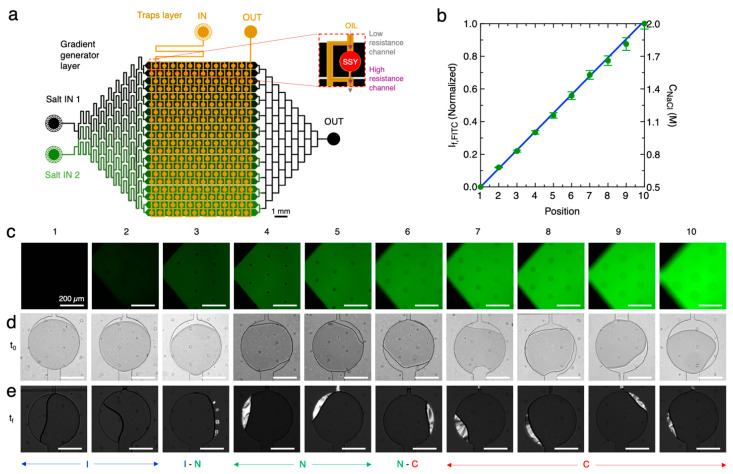
Incubation of SSY solutions in different phases by controlled drying. (**a**) Sketch of the microfluidic chip 3 inspired from ref. [62] composed of an upper layer hosting wells for trapping SSY droplets (orange) and a bottom layer designed to repetitively mix two input salt solutions to obtain a linear gradient of salt concentrations in the reservoir channels placed in correspondence with the traps (black). Inset shows the detail of a well placed at the bifurcation of the main channel in two channels with different hydrodynamic resistance, which allows the trapping of SSY droplets in oil. (**b**) Normalized fluorescence intensity, I_f_, measured in the reservoir channel during the mixing of 0.5 M and 2 M NaCl solutions, the latter doped with 1 mM FITC, as a function of the channel position. Data are reported as average values and error bars correspond to standard deviations of n = 5 measurements. The blue line represents the linear fit of data. (**c**) Fluorescence microscopy images of the initial part of the reservoir channels at different reservoir channel positions. (**d**) Bright-field microscopy images of traps loaded with SSY solution (initial concentration, c_SSY,0_ = 0.1 M) at the beginning of the experiment (t_0_). (**e**) Polarized microscopy images of the same traps t_f_ = 15 h, showing the reduction in droplet volume and the transition to different phases (I, N, C) and relative coexistence (I-N, N-C).

Based on the results reported in the previous section, we tested if the different liquid and liquid crystalline phases exhibited by SSY could be obtained as the final equilibrium condition of the drying process by using multiple reservoir channels containing different NaCl concentrations. To achieve this goal, we adopted a different two-layer chip (chip 3, Figure 5a), which was inspired by the “phase chip” reported in ref. [62]. Such a microfluidic device is composed of a 5 mm thick PDMS upper layer containing 240 circular wells (diameter 400 μm) located at bifurcations of the main channel (width 100 μm) and followed by a striction (width 25 μm). The height of the design is fixed (50 μm). Similar designs have been previously shown to allow the trapping of droplets when two immiscible fluids are subsequently fluxed from the main channel due to capillary forces and the different hydrodynamic resistance of the bifurcated channels in proximity to the circular traps [62,72]. The bottom thin PDMS layer is composed of a gradient generator obtained by the repetitive splitting and mixing of the flow of two aqueous fluids (a high-concentration NaCl solution and a low-concentration NaCl solution) in a branched network of channels. The chip is designed to produce a linear gradient of salt concentration in 10 parallel reservoir channels (height 50 μm and width 600 μm) placed in correspondence with the wells. Similarly to chip 2, water can diffuse through a thin PDMS membrane (50 μm) separating the wells and the reservoir layer driven by the difference in osmolarity of the two solutions.

We loaded the reservoir layer with 0.5 M and 2 M NaCl solutions, the latter doped with 1 mM fluorescein (FITC). The measurement of FITC fluorescence intensity, I_f_, at the entrance of the reservoir channels (Figure 5b,c) confirmed the linear distribution of salt concentration as a function of the channel position in the interval 0.5 < c_NaCl_ < 2 M.

**Figure 6:**
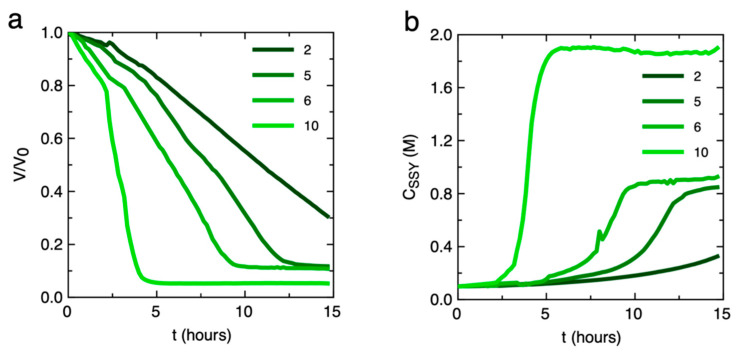
Plots of (**a**) the variation of normalized volume (V/V_0_) and (**b**) the SSY concentration (C_SSY_) over time of the droplets during incubation in chip 3. Volume was measured from a bright-field microscopy image sequence acquired at Δt = 10 min (Supplementary Materials). Curves correspond to droplets trapped in correspondence of channels 2, 5, 6, and 10 and thus exposed to a salt reservoir with increasing osmolarity. Droplets corresponding to higher osmolarity reservoir channels exhibit faster concentration and equilibration compared to those in lower osmolarity channels.

Then, the wells layer was initially loaded with a SSY solution, c_SSY,0_ = 0.1 M, which was followed by the fluxing of fluorinated oil with 2% surfactant allowing the trapping of equal concentration isotropic SSY droplets in the wells (Figure 5d). During the experiment time, the droplets started to equilibrate with the corresponding reservoir channels and reduced their volume. During time, as c_SSY_ was increasing, phase transitions between the SSY LC phases were observed as a function of the position of the wells (Figure 5e and Supplementary Materials). The variation of normalized droplet volume (V/V_0_) over time was measured from bright field microscopy images (Figure 6a), and the corresponding c_SSY_ was obtained (Figure 6b). It is possible to notice that on the side of the chip corresponding to reservoir channels with higher c_NaCl_, the shrinkage of the trapped SSY droplets was faster due to the high osmolarity difference between the SSY and NaCl solutions. Stable droplets exhibiting C, N-C, and N phases were obtained after 5, 10, and 13 h, respectively, indicating that an incubation of SSY solutions in different phases can be achieved.

## 4. Discussion

The reported experiments show that different microfluidics approaches can be used to investigate the phase diagram of SSY solutions as a function of concentration and temperature, to simulate drying–wetting cycles, and to incubate SSY solutions in different LC phases. These results were not granted, since the compatibility of microfluidic approaches based on water pervaporation through PDMS with solutions of small molecular weight molecules like SSY was not known before. Indeed, both an undesired permeation of fluid through PDMS walls [73] and the absorption of hydrophobic small molecules in PDMS [74], with possible alteration of measured concentrations, have been previously reported. We can disregard these effects as if they were present in our experiments, they would have led to an overestimation in the measurements of the SSY concentration, which contrasts with our actual observations.

Almost all the experiments reported here could have been in principle performed with just one microfluidic chip. Nevertheless, we found that the three tested microfluidic devices have both advantages and disadvantages to each other, which motivated the choice of using a different chip for each different application. We report the advantages and disadvantages in Table 1, hoping to offer the reader useful information for planning future experiments with these or similar devices.

A possible upgrade of chips 2 and 3 could be the addition of a glass or plastic layer above the upper PDMS layer to avoid water evaporation from the upper side of the chip.

## 5. Conclusions

Microfluidic platforms offer unparalleled advantages, enabling the manipulation of minute volumes of fluids with high precision, rapid mixing, and the capability to create controlled gradients. The integration of microfluidic systems with drying–wetting cycles represents a novel strategy to mimic dynamic environmental conditions relevant to early Earth, where fluctuating hydration states may have played a crucial role in prebiotic chemistry. By subjecting small molecules to controlled drying–wetting cycles within microfluidic channels, it can be possible to elucidate the impact of hydration fluctuations on the reversible formation of ordered phases typical of liquid crystals or other high-concentration phases, such as gels or glasses.

Leveraging the unique capabilities of microfluidics, including low sample consumption, low equilibration times, and automatization and parallelization possibilities, this approach can be extended to investigate the assembly dynamics of diverse small molecules solutions systematically, in particular nucleic acids monomers, pertinent to simulate prebiotic scenarios.

## Figures and Tables

**Table 1:** Advantages and disadvantages of the three microfluidic chips used in this work, which led us to the choice to use chip 1 for phase diagram investigation, chip 2 for implementing hydration–dehydration cycles, and chip 3 for the parallel incubation of samples in different phases.

	Advantages	Disadvantages
Chip 1	Fast and controlled production of droplets, large droplets surface-to-volume ratio that increases the sensitivity of the measurement of the volume.	Not compatible with a reservoir layer because the high deformability of the thin PDMS membrane causes surface tension and traps destabilization.
Chip 2	Fast and controlled production of droplets, compatibility of the trapping method with reservoir layer.	Lower droplets surface to volume ratio (i.e., less precision for phase diagram determination), too close traps and escaping of small (high concentration) droplets from traps (i.e., not suitable for parallel incubation of samples in different phases).
Chip 3	Compatible with multiple reservoir channels, large droplets surface to volume ratio.	Slower and less controlled droplets production process, non-circular shape of the droplets, possible splitting/escaping of the droplets during the drying process (i.e., less suitable for phase diagram determination).

**Figure A1:**
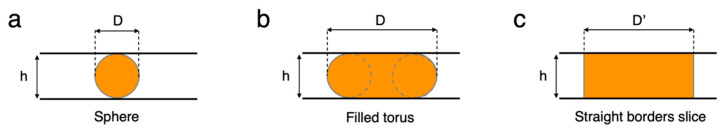
Volume of a droplet in a microfluidic channel. (**a**) Spere of D ≤ h. (**b**) Filled torus of D > h. (**c**) Straight border slice of D′ >> h.

## Data Availability

The data presented in this study and high-resolution lithography masks files are available on request from the corresponding author.

## Supplementary Materials

The following supporting information can be downloaded at: https://www.mdpi.com/article/10.3390/life14040472/s1, Python code for droplets images analysis, lithography masks of the microfluidic chips, and supplementary figures.

## Appendix A. Calculation of the Volume of a Droplet in the Microfluidic Device

The shape of a droplet in the microfluidic chips 1 and 2 can be assumed as a sphere only if the droplet diameter is smaller than the height of the confining channel or trap, D ≤ h (Figure A1a). In this case, the volume of the droplet is the volume of the sphere:

V = 1/6 πD^3^, (A1)

Otherwise, if the diameter is larger than the height of the channel, D > h, the droplet is confined, its shape is squeezed and its volume can be calculated with good approximation as the volume of a filled torus of diameter D (Figure A1b) [61]:

V = 1/4 πhD^3^ (1 − 2 h + 5/3 h^2^ + π/2 h (1 − h)), (A2)

To calculate the volume of the SSY droplets from the measurement of their apparent diameter, we used Equations (A1) and (A2) according to the value of D with respect to h.

For chip 3, since the shapes of the trapped droplets were non-circular and were changing over time, the volume was calculated as

V = Ah, (A3)

where A is the surface of the droplet and h is the height of the trap. This means that the droplet is considered as a slice of surface A with straight borders (Figure A1c). This approximation, which does not consider the droplet curvature at the border, is acceptable due to the large area-to-height ratio of the droplets, i.e., D′ >> h (where D is of the order of A^1/2^).
