# Supplementary material for: Microfluidics-Based Drying–Wetting Cycles to Investigate Phase Transitions of Small Molecules Solutions
Source: Life (Basel). 2024 Apr 4;14(4):472. doi: 10.3390/life14040472 (PMC11050796; doi:10.3390/life14040472)
Supplement: Supplementary file 1 [file life-14-00472-s001.zip › Supplementary Material - Life.pdf]

Microfluidics Based Drying-Wetting Cycles to Investigate Phase Transitions of Small Molecules Solutions

Ajay Verma [1], Tiphaine Mateo [1], Juan Quintero Botero [2], Nishanth Mohankumar [1] and Tommaso P. Fraccia [1,3,*]

[1] IPGG, CBI UMR 8231 – CNRS – ESPCI Paris, PSL Research University, 75005 Paris, France
[2] Department of Chemistry, Columbia University, New York, NY 10027, USA
[3] Department of Pharmacological and Biomolecular Sciences, University of Milan, 20133 Milan, Italy
* Correspondence: tommaso.fraccia@unimi.it

Supplementary Material

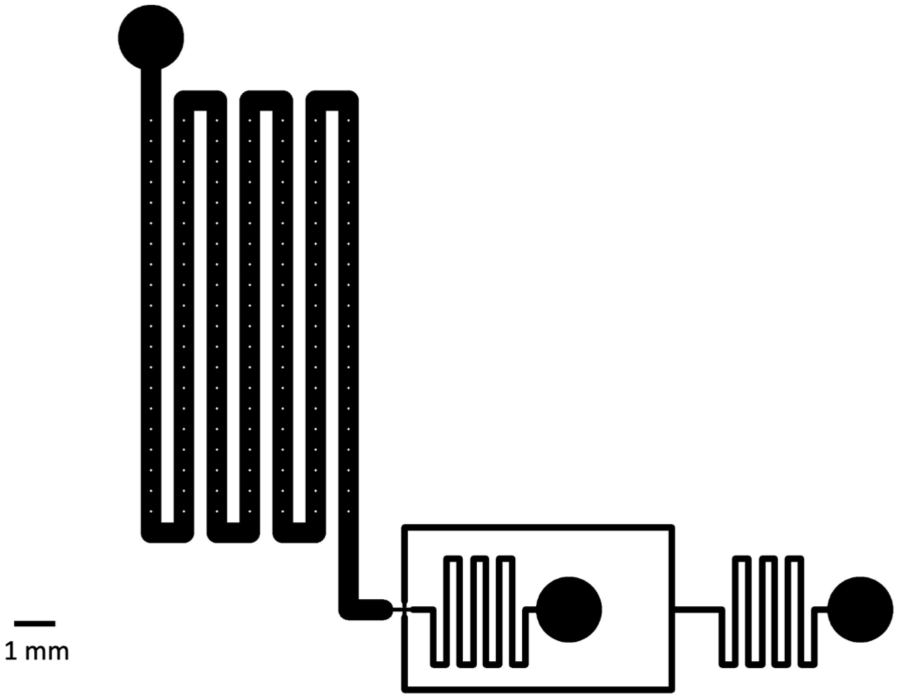

Supplementary Figure S1. Lithography mask of chip 1: single layer chip, flow focusing and traps.

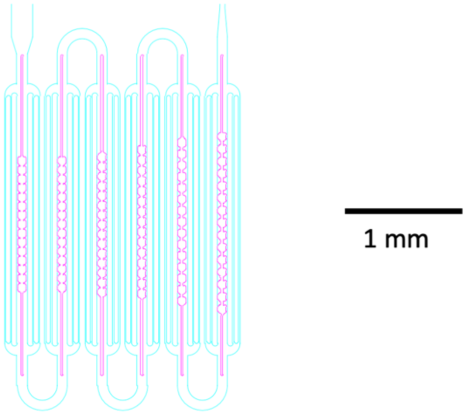

Supplementary Figure S2. Lithography mask of chip 2: upper layer – channels and traps.

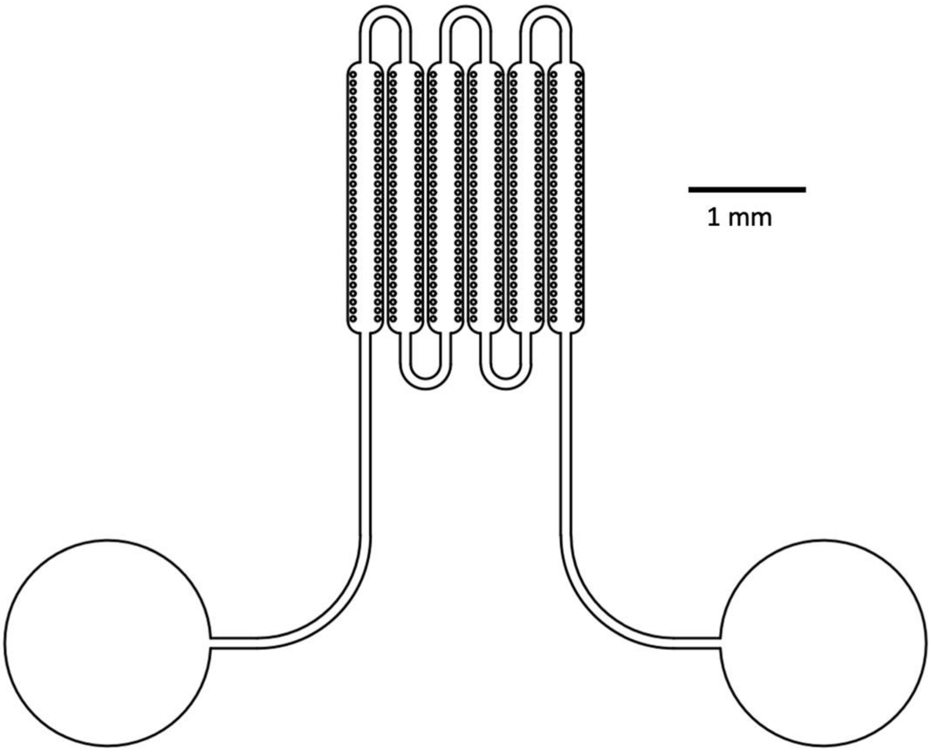

Supplementary Figure S3. Lithography mask of chip 2: bottom layer – reservoir.

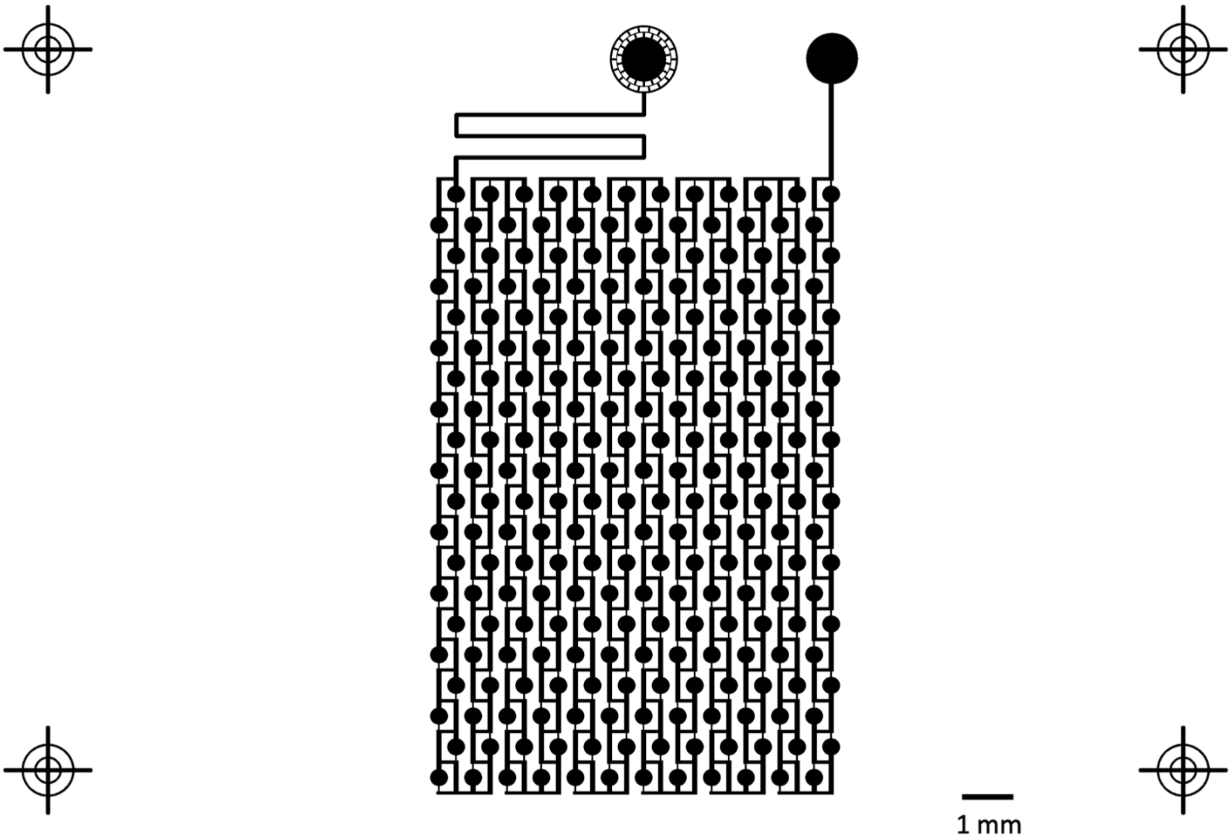

Supplementary Figure S5. Lithography mask of chip 3: upper layer – traps.

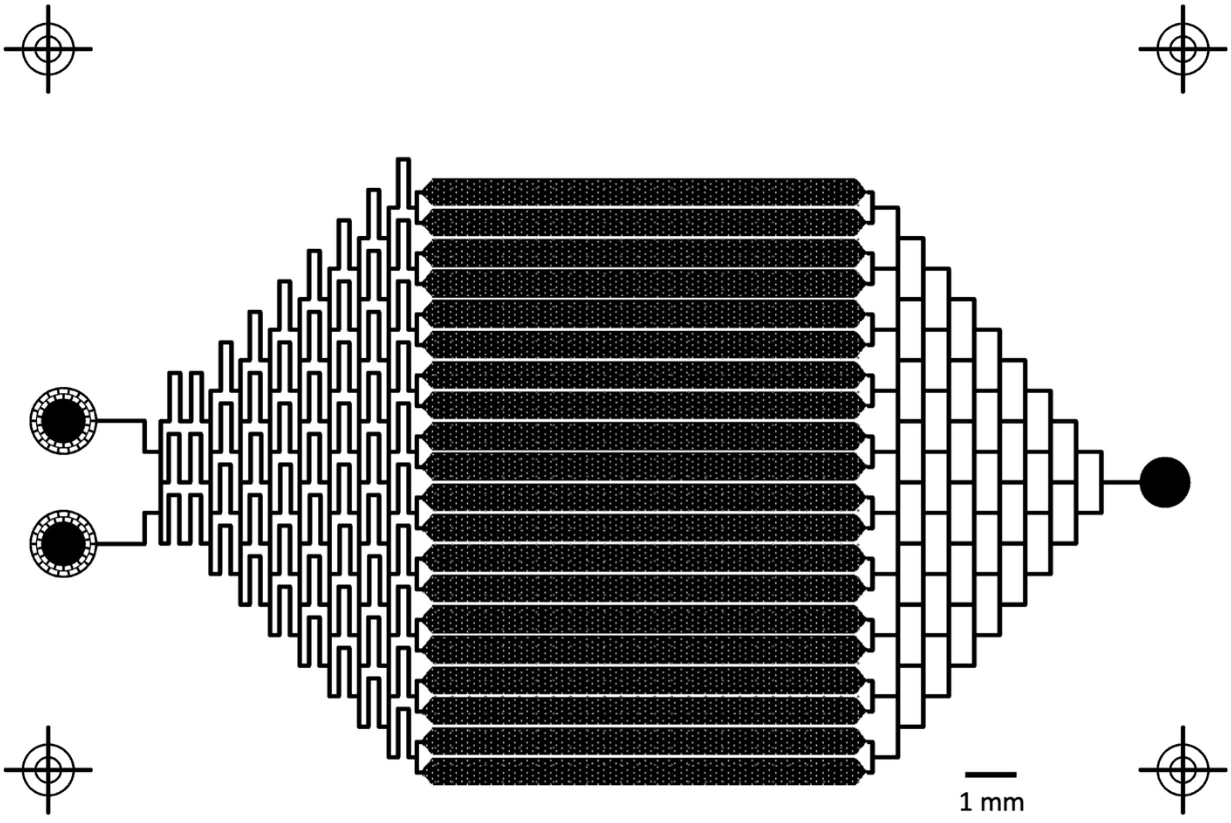

Supplementary Figure S6. Lithography mask of chip 3: bottom layer – reservoir.

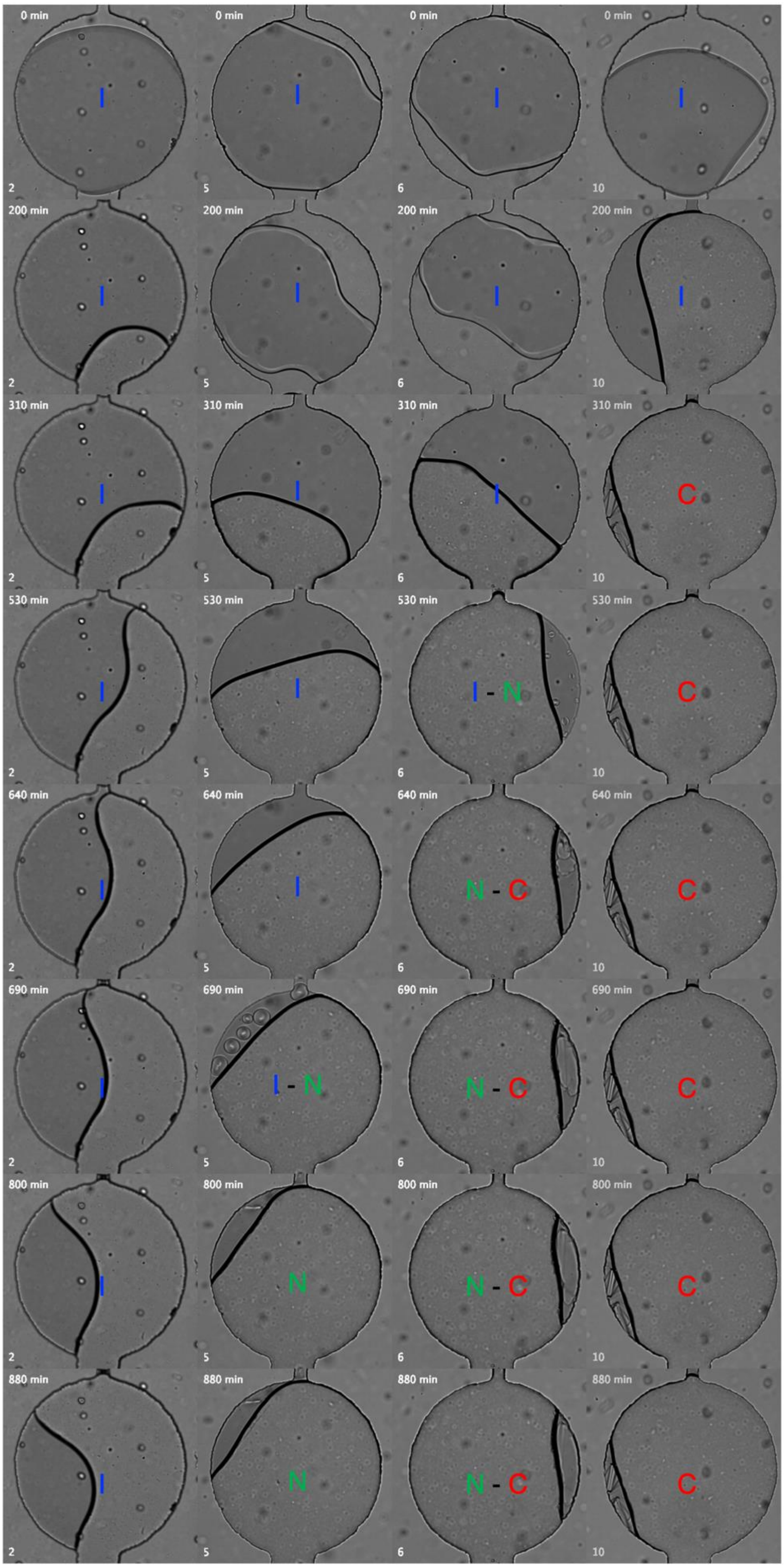

Supplementary Figure S7. Bright field microscopy images at different time steps of SSY solutions incubated within chip 3 in correspondence to reservoir channels 2, 5, 6 and 10 (from left to right). Phases identification are reported (I, I - N, N, N - C, C).
